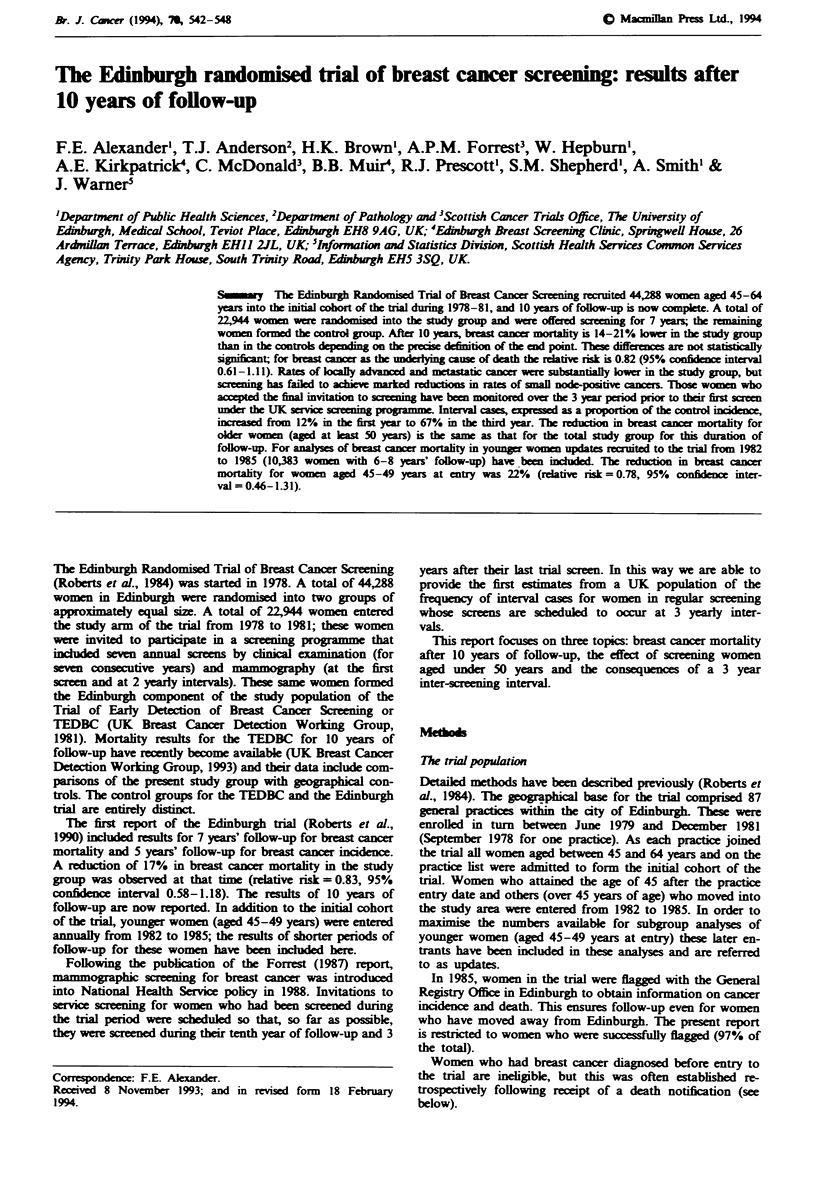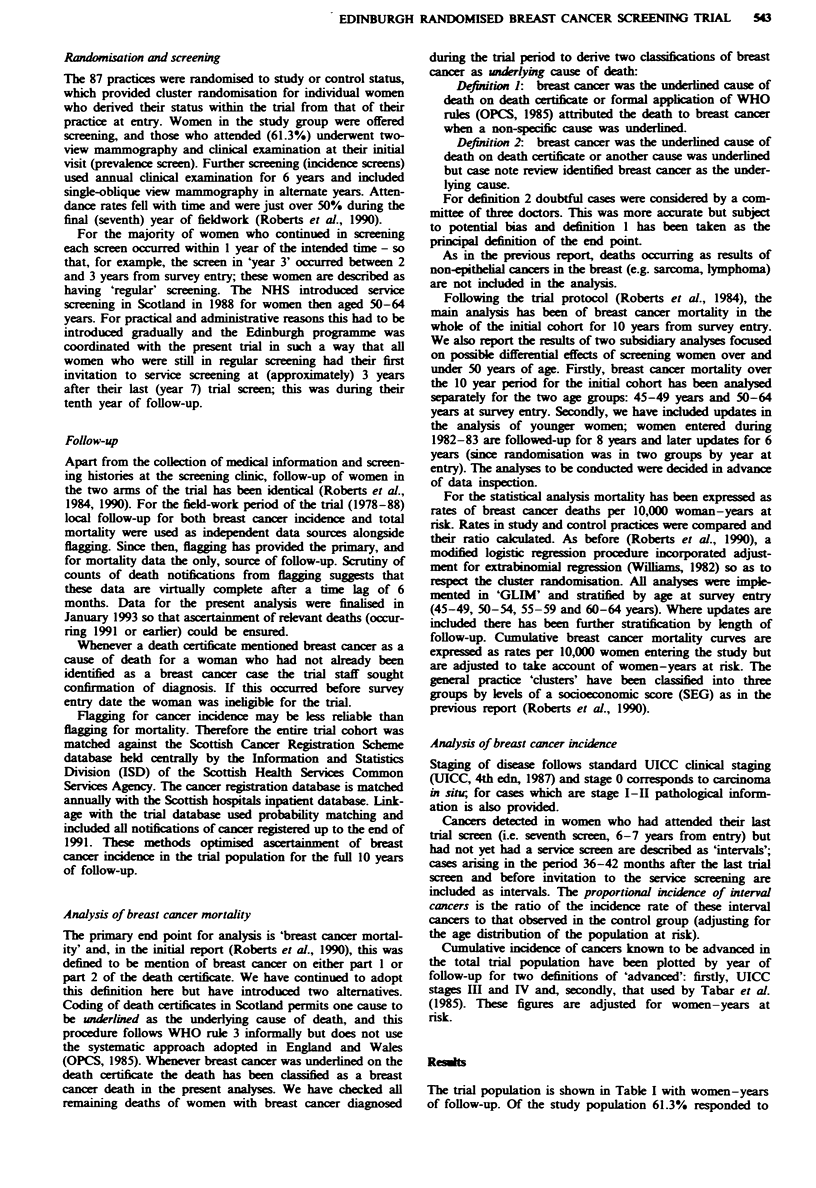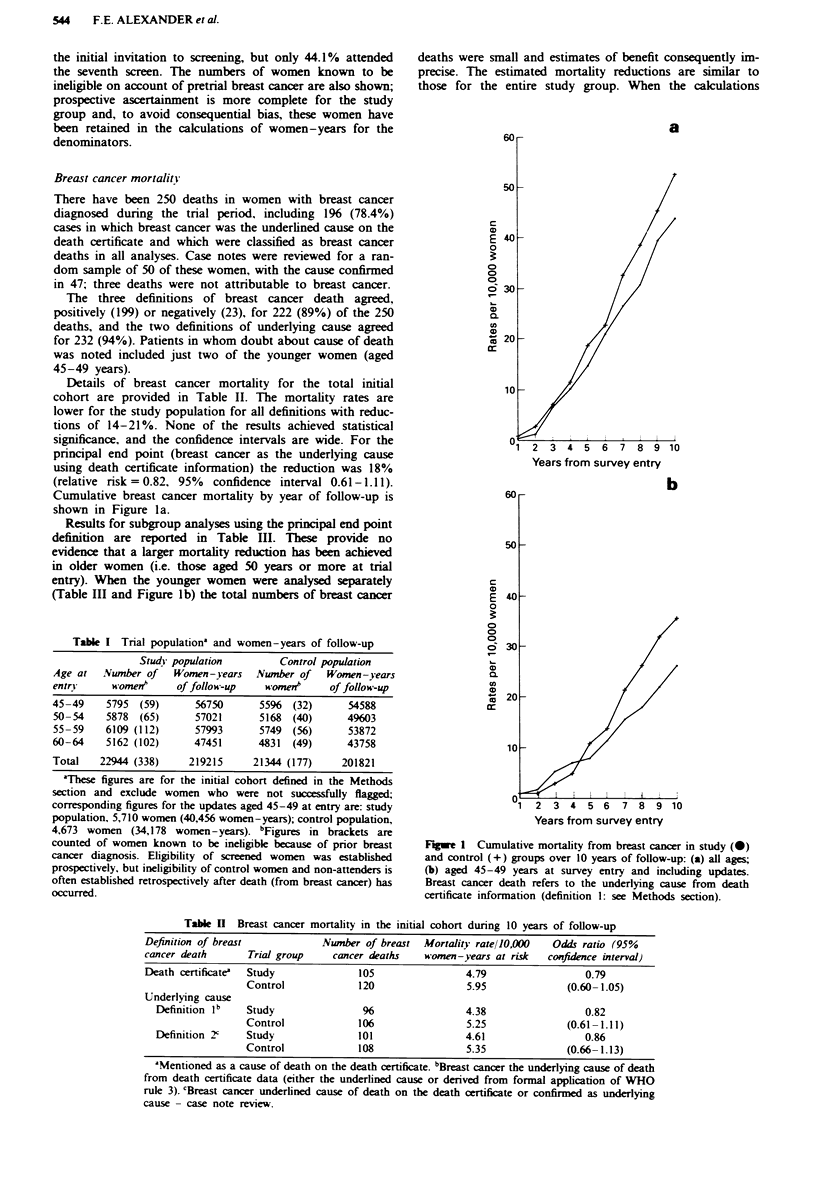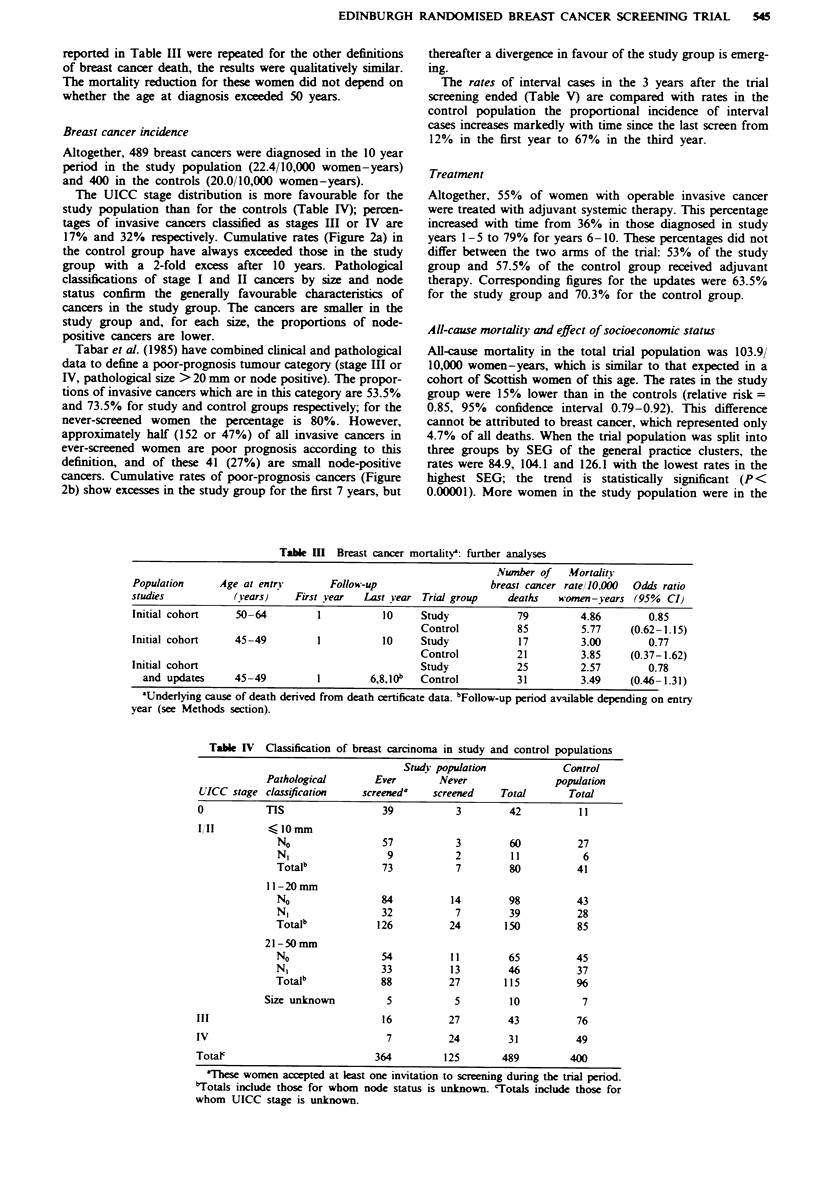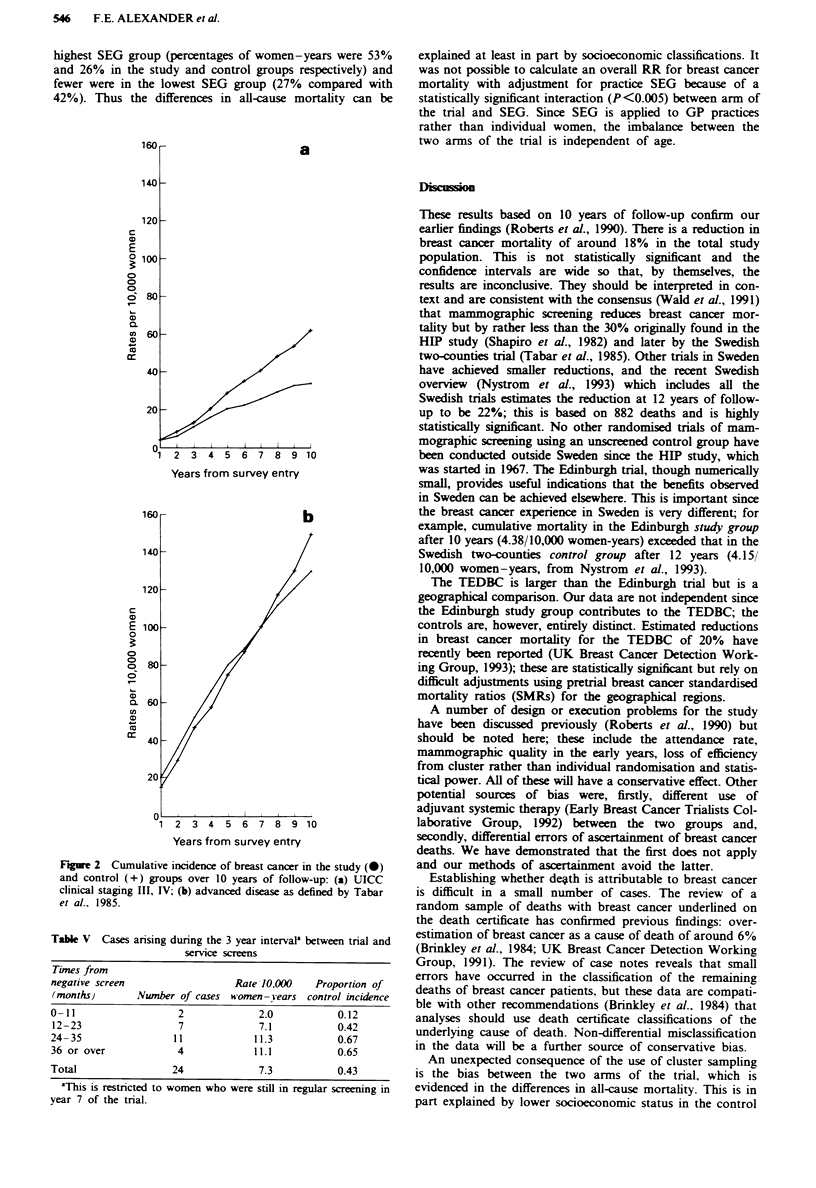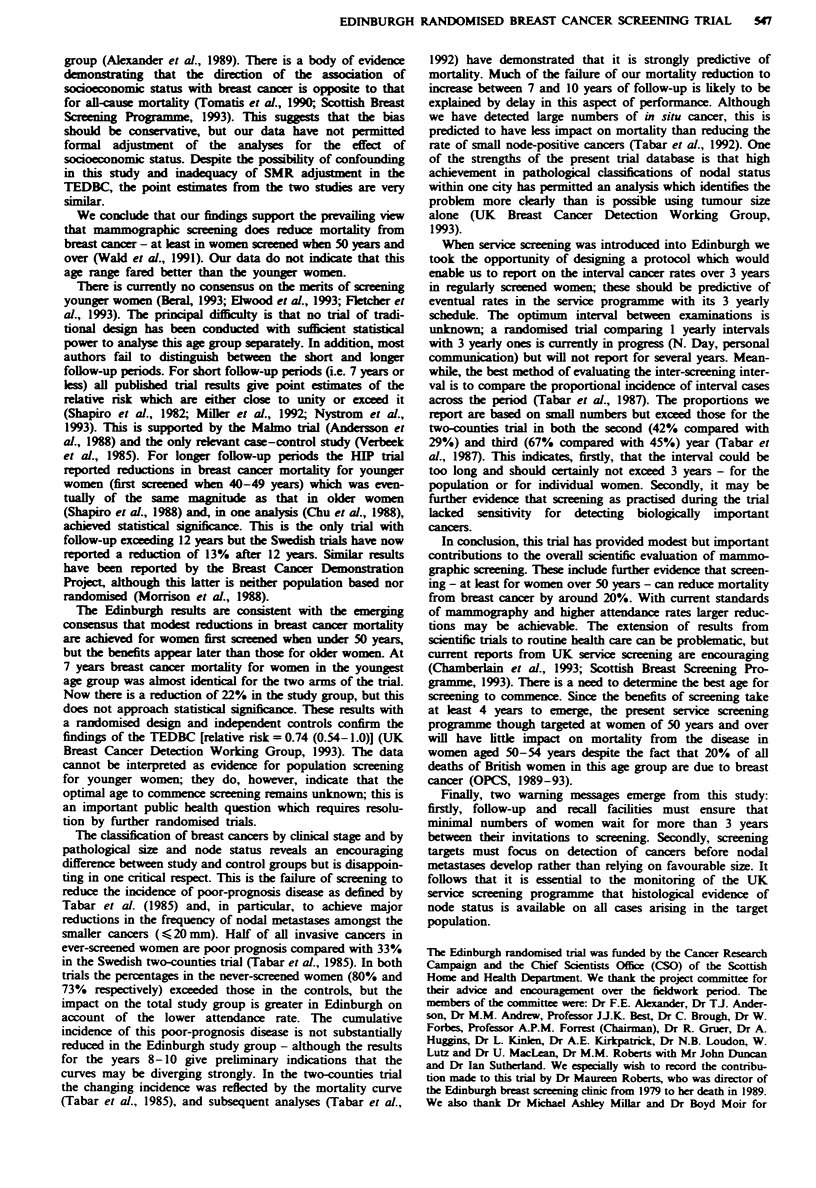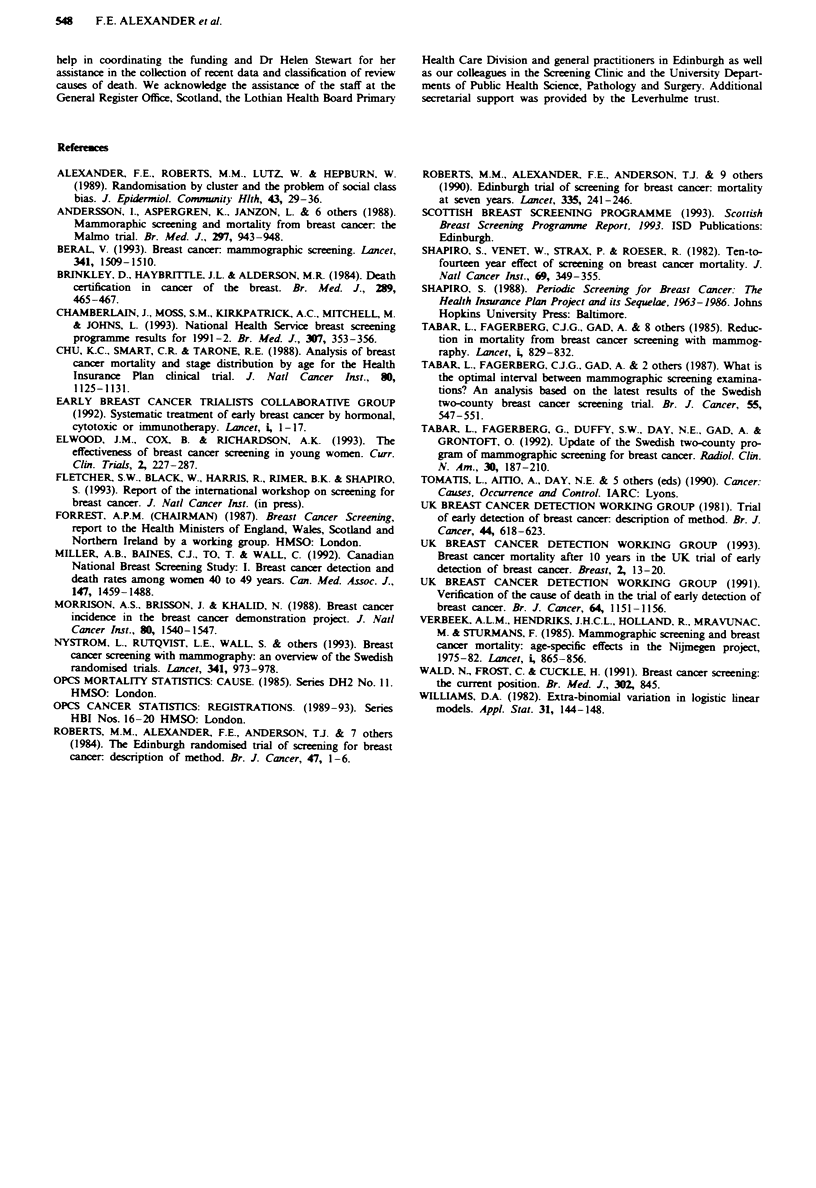# The Edinburgh randomised trial of breast cancer screening: results after 10 years of follow-up.

**DOI:** 10.1038/bjc.1994.342

**Published:** 1994-09

**Authors:** F. E. Alexander, T. J. Anderson, H. K. Brown, A. P. Forrest, W. Hepburn, A. E. Kirkpatrick, C. McDonald, B. B. Muir, R. J. Prescott, S. M. Shepherd

**Affiliations:** Department of Public Health Sciences, University of Edinburgh, Medical School, UK.

## Abstract

The Edinburgh Randomised Trial of Breast Cancer Screening recruited 44,288 women aged 45-64 years into the initial cohort of the trial during 1978-81, and 10 years of follow-up is now complete. A total of 22,944 women were randomised into the study group and were offered screening for 7 years; the remaining women formed the control group. After 10 years, breast cancer mortality is 14-21% lower in the study group than in the controls depending on the precise definition of the end point. These differences are not statistically significant; for breast cancer as the underlying cause of death the relative risk is 0.82 (95% confidence interval 0.61-1.11). Rates of locally advanced and metastatic cancer were substantially lower in the study group, but screening has failed to achieve marked reductions in rates of small node-positive cancers. Those women who accepted the final invitation to screening have been monitored over the 3 year period prior to their first screen under the UK service screening programme. Interval cases, expressed as a proportion of the control incidence, increased from 12% in the first year to 67% in the third year. The reduction in breast cancer mortality for older women (aged at least 50 years) is the same as that for the total study group for this duration of follow-up. For analyses of breast cancer mortality in younger women updates recruited to the trial from 1982 to 1985 (10,383 women with 6-8 years' follow-up) have been included. The reduction in breast cancer mortality for women aged 45-49 years at entry was 22% (relative risk = 0.78, 95% confidence interval = 0.46-1.31).


### The Edinburgh randomised trial of breast cancer screening: results after 10 years of follow-up

F.E. Alexander[1], T.J. Anderson[2], H.K. Brown[1], A.P.M. Forrest[3], W. Hepburn[1], A.E. Kirkpatrick[4], C. McDonald[3], B.B. Muir[4], R.J. Prescott[1], S.M. Shepherd[1], A. Smith[1] & J. Warner[5]

[1]*Department of Public Health Sciences,* [2]*Department of Pathology and* [3]*Scottish Cancer Trials Office, The University of Edinburgh, Medical School, Teviot Place, Edinburgh EH8 9AG, UK;* [4]*Edinburgh Breast Screening Clinic, Springwell House, 26 Ardmillan Terrace, Edinburgh EH11 2JL, UK;* [5]*Information and Statistics Division, Scottish Health Services Common Services Agency, Trinity Park House, South Trinity Road, Edinburgh EH5 3SQ, UK.*

**Summary** The Edinburgh Randomised Trial of Breast Cancer Screening recruited 44,288 women aged 45-64 years into the initial cohort of the trial during 1978-81, and 10 years of follow-up is now complete. A total of 22,944 women were randomised into the study group and were offered screening for 7 years; the remaining women formed the control group. After 10 years, breast cancer mortality is 14-21% lower in the study group than in the controls depending on the precise definition of the end point. These differences are not statistically significant; for breast cancer as the underlying cause of death the relative risk is 0.82 (95% confidence interval 0.61-1.11). Rates of locally advanced and metastatic cancer were substantially lower in the study group, but screening has failed to achieve marked reductions in rates of small node-positive cancers. Those women who accepted the final invitation to screening have been monitored over the 3 year period prior to their first screen under the UK service screening programme. Interval cases, expressed as a proportion of the control incidence, increased from 12% in the first year to 67% in the third year. The reduction in breast cancer mortality for older women (aged at least 50 years) is the same as that for the total study group for this duration of follow-up. For analyses of breast cancer mortality in younger women updates recruited to the trial from 1982 to 1985 (10,383 women with 6-8 years' follow-up) have been included. The reduction in breast cancer mortality for women aged 45-49 years at entry was 22% (relative risk = 0.78, 95% confidence interval = 0.46-1.31).

The Edinburgh Randomised Trial of Breast Cancer Screening (Roberts *et al.*, 1984) was started in 1978. A total of 44,288 women in Edinburgh were randomised into two groups of approximately equal size. A total of 22,944 women entered the study arm of the trial from 1978 to 1981; these women were invited to participate in a screening programme that included seven annual screens by clinical examination (for seven consecutive years) and mammography (at the first screen and at 2 yearly intervals). These same women formed the Edinburgh component of the study population of the Trial of Early Detection of Breast Cancer Screening or TEDBC (UK Breast Cancer Detection Working Group, 1981). Mortality results for the TEDBC for 10 years of follow-up have recently become available (UK Breast Cancer Detection Working Group, 1993) and their data include comparisons of the present study group with geographical controls. The control groups for the TEDBC and the Edinburgh trial are entirely distinct.

The first report of the Edinburgh trial (Roberts *et al.*, 1990) included results for 7 years' follow-up for breast cancer mortality and 5 years' follow-up for breast cancer incidence. A reduction of 17% in breast cancer mortality in the study group was observed at that time (relative risk = 0.83, 95% confidence interval 0.58-1.18). The results of 10 years of follow-up are now reported. In addition to the initial cohort of the trial, younger women (aged 45-49 years) were entered annually from 1982 to 1985; the results of shorter periods of follow-up for these women have been included here.

Following the publication of the Forrest (1987) report, mammographic screening for breast cancer was introduced into National Health Service policy in 1988. Invitations to service screening for women who had been screened during the trial period were scheduled so that, so far as possible, they were screened during their tenth year of follow-up and 3 years after their last trial screen. In this way we are able to provide the first estimates from a UK population of the frequency of interval cases for women in regular screening whose screens are scheduled to occur at 3 yearly intervals.

This report focuses on three topics: breast cancer mortality after 10 years of follow-up, the effect of screening women aged under 50 years and the consequences of a 3 year inter-screening interval.

### Methods

### *The trial population*

Detailed methods have been described previously (Roberts *et al.*, 1984). The geographical base for the trial comprised 87 general practices within the city of Edinburgh. These were enrolled in turn between June 1979 and December 1981 (September 1978 for one practice). As each practice joined the trial all women aged between 45 and 64 years and on the practice list were admitted to form the initial cohort of the trial. Women who attained the age of 45 after the practice entry date and others (over 45 years of age) who moved into the study area were entered from 1982 to 1985. In order to maximise the numbers available for subgroup analyses of younger women (aged 45-49 years at entry) these later entrants have been included in these analyses and are referred to as updates.

In 1985, women in the trial were flagged with the General Registry Office in Edinburgh to obtain information on cancer incidence and death. This ensures follow-up even for women who have moved away from Edinburgh. The present report is restricted to women who were successfully flagged (97% of the total).

Women who had breast cancer diagnosed before entry to the trial are ineligible, but this was often established retrospectively following receipt of a death notification (see below).

Correspondence: F.E. Alexander.
Received 8 November 1993; and in revised form 18 February 1994.

*Randomisation and screening*

The 87 practices were randomised to study or control status, which provided cluster randomisation for individual women who derived their status within the trial from that of their practice at entry. Women in the study group were offered screening, and those who attended (61.3%) underwent two-view mammography and clinical examination at their initial visit (prevalence screen). Further screening (incidence screens) used annual clinical examination for 6 years and included single-oblique view mammography in alternate years. Attendance rates fell with time and were just over 50% during the final (seventh) year of fieldwork (Roberts *et al.*, 1990).

For the majority of women who continued in screening each screen occurred within 1 year of the intended time - so that, for example, the screen in 'year 3' occurred between 2 and 3 years from survey entry; these women are described as having 'regular' screening. The NHS introduced service screening in Scotland in 1988 for women then aged 50-64 years. For practical and administrative reasons this had to be introduced gradually and the Edinburgh programme was coordinated with the present trial in such a way that all women who were still in regular screening had their first invitation to service screening at (approximately) 3 years after their last (year 7) trial screen; this was during their tenth year of follow-up.

*Follow-up*

Apart from the collection of medical information and screening histories at the screening clinic, follow-up of women in the two arms of the trial has been identical (Roberts *et al.*, 1984, 1990). For the field-work period of the trial (1978-88) local follow-up for both breast cancer incidence and total mortality were used as independent data sources alongside flagging. Since then, flagging has provided the primary, and for mortality data the only, source of follow-up. Scrutiny of counts of death notifications from flagging suggests that these data are virtually complete after a time lag of 6 months. Data for the present analysis were finalised in January 1993 so that ascertainment of relevant deaths (occurring 1991 or earlier) could be ensured.

Whenever a death certificate mentioned breast cancer as a cause of death for a woman who had not already been identified as a breast cancer case the trial staff sought confirmation of diagnosis. If this occurred before survey entry date the woman was ineligible for the trial.

Flagging for cancer incidence may be less reliable than flagging for mortality. Therefore the entire trial cohort was matched against the Scottish Cancer Registration Scheme database held centrally by the Information and Statistics Division (ISD) of the Scottish Health Services Common Services Agency. The cancer registration database is matched annually with the Scottish hospitals inpatient database. Linkage with the trial database used probability matching and included all notifications of cancer registered up to the end of 1991. These methods optimised ascertainment of breast cancer incidence in the trial population for the full 10 years of follow-up.

*Analysis of breast cancer mortality*

The primary end point for analysis is 'breast cancer mortality' and, in the initial report (Roberts *et al.*, 1990), this was defined to be mention of breast cancer on either part 1 or part 2 of the death certificate. We have continued to adopt this definition here but have introduced two alternatives. Coding of death certificates in Scotland permits one cause to be *underlined* as the underlying cause of death, and this procedure follows WHO rule 3 informally but does not use the systematic approach adopted in England and Wales (OPCS, 1985). Whenever breast cancer was underlined on the death certificate the death has been classified as a breast cancer death in the present analyses. We have checked all remaining deaths of women with breast cancer diagnosed during the trial period to derive two classifications of breast cancer as *underlying* cause of death:

*Definition 1*: breast cancer was the underlined cause of death on death certificate or formal application of WHO rules (OPCS, 1985) attributed the death to breast cancer when a non-specific cause was underlined.

*Definition 2*: breast cancer was the underlined cause of death on death certificate or another cause was underlined but case note review identified breast cancer as the underlying cause.

For definition 2 doubtful cases were considered by a committee of three doctors. This was more accurate but subject to potential bias and definition 1 has been taken as the principal definition of the end point.

As in the previous report, deaths occurring as results of non-epithelial cancers in the breast (e.g. sarcoma, lymphoma) are not included in the analysis.

Following the trial protocol (Roberts *et al.*, 1984), the main analysis has been of breast cancer mortality in the whole of the initial cohort for 10 years from survey entry. We also report the results of two subsidiary analyses focused on possible differential effects of screening women over and under 50 years of age. Firstly, breast cancer mortality over the 10 year period for the initial cohort has been analysed separately for the two age groups: 45-49 years and 50-64 years at survey entry. Secondly, we have included updates in the analysis of younger women; women entered during 1982-83 are followed-up for 8 years and later updates for 6 years (since randomisation was in two groups by year at entry). The analyses to be conducted were decided in advance of data inspection.

For the statistical analysis mortality has been expressed as rates of breast cancer deaths per 10,000 woman-years at risk. Rates in study and control practices were compared and their ratio calculated. As before (Roberts *et al.*, 1990), a modified logistic regression procedure incorporated adjustment for extrabinomial regression (Williams, 1982) so as to respect the cluster randomisation. All analyses were implemented in 'GLIM' and stratified by age at survey entry (45-49, 50-54, 55-59 and 60-64 years). Where updates are included there has been further stratification by length of follow-up. Cumulative breast cancer mortality curves are expressed as rates per 10,000 women entering the study but are adjusted to take account of women-years at risk. The general practice 'clusters' have been classified into three groups by levels of a socioeconomic score (SEG) as in the previous report (Roberts *et al.*, 1990).

*Analysis of breast cancer incidence*

Staging of disease follows standard UICC clinical staging (UICC, 4th edn, 1987) and stage 0 corresponds to carcinoma *in situ*; for cases which are stage I-II pathological information is also provided.

Cancers detected in women who had attended their last trial screen (i.e. seventh screen, 6-7 years from entry) but had not yet had a service screen are described as 'intervals'; cases arising in the period 36-42 months after the last trial screen and before invitation to the service screening are included as intervals. The *proportional incidence of interval cancers* is the ratio of the incidence rate of these interval cancers to that observed in the control group (adjusting for the age distribution of the population at risk).

Cumulative incidence of cancers known to be advanced in the total trial population have been plotted by year of follow-up for two definitions of 'advanced': firstly, UICC stages III and IV and, secondly, that used by Tabar *et al.* (1985). These figures are adjusted for women-years at risk.

### Results

The trial population is shown in Table I with women-years of follow-up. Of the study population 61.3% responded to the initial invitation to screening, but only 44.1% attended the seventh screen. The numbers of women known to be ineligible on account of pretrial breast cancer are also shown; prospective ascertainment is more complete for the study group and, to avoid consequential bias, these women have been retained in the calculations of women-years for the denominators.

*Breast cancer mortality*

There have been 250 deaths in women with breast cancer diagnosed during the trial period, including 196 (78.4%) cases in which breast cancer was the underlined cause on the death certificate and which were classified as breast cancer deaths in all analyses. Case notes were reviewed for a random sample of 50 of these women, with the cause confirmed in 47; three deaths were not attributable to breast cancer.

The three definitions of breast cancer death agreed, positively (199) or negatively (23), for 222 (89%) of the 250 deaths, and the two definitions of underlying cause agreed for 232 (94%). Patients in whom doubt about cause of death was noted included just two of the younger women (aged 45-49 years).

Details of breast cancer mortality for the total initial cohort are provided in Table II. The mortality rates are lower for the study population for all definitions with reductions of 14-21%. None of the results achieved statistical significance, and the confidence intervals are wide. For the principal end point (breast cancer as the underlying cause using death certificate information) the reduction was 18% (relative risk = 0.82, 95% confidence interval 0.61-1.11). Cumulative breast cancer mortality by year of follow-up is shown in Figure 1a.

Results for subgroup analyses using the principal end point definition are reported in Table III. These provide no evidence that a larger mortality reduction has been achieved in older women (i.e. those aged 50 years or more at trial entry). When the younger women were analysed separately (Table III and Figure 1b) the total numbers of breast cancer deaths were small and estimates of benefit consequently imprecise. The estimated mortality reductions are similar to those for the entire study group. When the calculations

**Figure 1** Cumulative mortality from breast cancer in study (●) and control (+) groups over 10 years of follow-up: (**a**) all ages; (**b**) aged 45-49 years at survey entry and including updates. Breast cancer death refers to the underlying cause from death certificate information (definition 1: see Methods section).

**Table I** Trial population[a] and women-years of follow-up

| | *Study population* | | *Control population* | |
|---|---|---|---|---|
| *Age at entry* | *Number of women*[b] | *Women-years of follow-up* | *Number of women*[b] | *Women-years of follow-up* |
| 45-49 | 5795 (59) | 56750 | 5596 (32) | 54588 |
| 50-54 | 5878 (65) | 57021 | 5168 (40) | 49603 |
| 55-59 | 6109 (112) | 57993 | 5749 (56) | 53872 |
| 60-64 | 5162 (102) | 47451 | 4831 (49) | 43758 |
| Total | 22944 (338) | 219215 | 21344 (177) | 201821 |

[a]These figures are for the initial cohort defined in the Methods section and exclude women who were not successfully flagged; corresponding figures for the updates aged 45-49 at entry are: study population, 5,710 women (40,456 women-years); control population, 4,673 women (34,178 women-years). [b]Figures in brackets are counted of women known to be ineligible because of prior breast cancer diagnosis. Eligibility of screened women was established prospectively, but ineligibility of control women and non-attenders is often established retrospectively after death (from breast cancer) has occurred.

**Table II** Breast cancer mortality in the initial cohort during 10 years of follow-up

| *Definition of breast cancer death* | *Trial group* | *Number of breast cancer deaths* | *Mortality rate/10,000 women-years at risk* | *Odds ratio (95% confidence interval)* |
|---|---|---|---|---|
| Death certificate[a] | Study | 105 | 4.79 | 0.79 |
| | Control | 120 | 5.95 | (0.60-1.05) |
| Underlying cause | | | | |
| Definition 1[b] | Study | 96 | 4.38 | 0.82 |
| | Control | 106 | 5.25 | (0.61-1.11) |
| Definition 2[c] | Study | 101 | 4.61 | 0.86 |
| | Control | 108 | 5.35 | (0.66-1.13) |

[a]Mentioned as a cause of death on the death certificate. [b]Breast cancer the underlying cause of death from death certificate data (either the underlined cause or derived from formal application of WHO rule 3). [c]Breast cancer underlined cause of death on the death certificate or confirmed as underlying cause - case note review.

reported in Table III were repeated for the other definitions of breast cancer death, the results were qualitatively similar. The mortality reduction for these women did not depend on whether the age at diagnosis exceeded 50 years.

*Breast cancer incidence*

Altogether, 489 breast cancers were diagnosed in the 10 year period in the study population (22.4/10,000 women-years) and 400 in the controls (20.0/10,000 women-years).

The UICC stage distribution is more favourable for the study population than for the controls (Table IV); percentages of invasive cancers classified as stages III or IV are 17% and 32% respectively. Cumulative rates (Figure 2a) in the control group have always exceeded those in the study group with a 2-fold excess after 10 years. Pathological classifications of stage I and II cancers by size and node status confirm the generally favourable characteristics of cancers in the study group. The cancers are smaller in the study group and, for each size, the proportions of node-positive cancers are lower.

Tabar *et al.* (1985) have combined clinical and pathological data to define a poor-prognosis tumour category (stage III or IV, pathological size >20 mm or node positive). The proportions of invasive cancers which are in this category are 53.5% and 73.5% for study and control groups respectively; for the never-screened women the percentage is 80%. However, approximately half (152 or 47%) of all invasive cancers in ever-screened women are poor prognosis according to this definition, and of these 41 (27%) are small node-positive cancers. Cumulative rates of poor-prognosis cancers (Figure 2b) show excesses in the study group for the first 7 years, but thereafter a divergence in favour of the study group is emerging.

The *rates* of interval cases in the 3 years after the trial screening ended (Table V) are compared with rates in the control population the proportional incidence of interval cases increases markedly with time since the last screen from 12% in the first year to 67% in the third year.

*Treatment*

Altogether, 55% of women with operable invasive cancer were treated with adjuvant systemic therapy. This percentage increased with time from 36% in those diagnosed in study years 1-5 to 79% for years 6-10. These percentages did not differ between the two arms of the trial: 53% of the study group and 57.5% of the control group received adjuvant therapy. Corresponding figures for the updates were 63.5% for the study group and 70.3% for the control group.

*All-cause mortality and effect of socioeconomic status*

All-cause mortality in the total trial population was 103.9/10,000 women-years, which is similar to that expected in a cohort of Scottish women of this age. The rates in the study group were 15% lower than in the controls (relative risk = 0.85, 95% confidence interval 0.79-0.92). This difference cannot be attributed to breast cancer, which represented only 4.7% of all deaths. When the trial population was split into three groups by SEG of the general practice clusters, the rates were 84.9, 104.1 and 126.1 with the lowest rates in the highest SEG; the trend is statistically significant ($P <$ 0.00001). More women in the study population were in the

**Table III** Breast cancer mortality[a]: further analyses

| *Population studies* | *Age at entry (years)* | *Follow-up First year* | *Follow-up Last year* | *Trial group* | *Number of breast cancer deaths* | *Mortality rate/10,000 women-years* | *Odds ratio (95% CI)* |
|---|---|---|---|---|---|---|---|
| Initial cohort | 50-64 | 1 | 10 | Study | 79 | 4.86 | 0.85 |
| | | | | Control | 85 | 5.77 | (0.62-1.15) |
| Initial cohort | 45-49 | 1 | 10 | Study | 17 | 3.00 | 0.77 |
| | | | | Control | 21 | 3.85 | (0.37-1.62) |
| Initial cohort | | | | Study | 25 | 2.57 | 0.78 |
| and updates | 45-49 | 1 | 6,8,10[b] | Control | 31 | 3.49 | (0.46-1.31) |

[a]Underlying cause of death derived from death certificate data. [b]Follow-up period available depending on entry year (see Methods section).

**Table IV** Classification of breast carcinoma in study and control populations

| *UICC stage* | *Pathological classification* | *Study population Ever screened*[a] | *Study population Never screened* | *Total* | *Control population Total* |
|---|---|---|---|---|---|
| 0 | TIS | 39 | 3 | 42 | 11 |
| I/II | ≤10 mm | | | | |
| | $N_0$ | 57 | 3 | 60 | 27 |
| | $N_1$ | 9 | 2 | 11 | 6 |
| | Total[b] | 73 | 7 | 80 | 41 |
| | 11-20 mm | | | | |
| | $N_0$ | 84 | 14 | 98 | 43 |
| | $N_1$ | 32 | 7 | 39 | 28 |
| | Total[b] | 126 | 24 | 150 | 85 |
| | 21-50 mm | | | | |
| | $N_0$ | 54 | 11 | 65 | 45 |
| | $N_1$ | 33 | 13 | 46 | 37 |
| | Total[b] | 88 | 27 | 115 | 96 |
| | Size unknown | 5 | 5 | 10 | 7 |
| III | | 16 | 27 | 43 | 76 |
| IV | | 7 | 24 | 31 | 49 |
| Total[c] | | 364 | 125 | 489 | 400 |

[a]These women accepted at least one invitation to screening during the trial period. [b]Totals include those for whom node status is unknown. [c]Totals include those for whom UICC stage is unknown.

highest SEG group (percentages of women-years were 53% and 26% in the study and control groups respectively) and fewer were in the lowest SEG group (27% compared with 42%). Thus the differences in all-cause mortality can be explained at least in part by socioeconomic classifications. It was not possible to calculate an overall RR for breast cancer mortality with adjustment for practice SEG because of a statistically significant interaction ($P<0.005$) between arm of the trial and SEG. Since SEG is applied to GP practices rather than individual women, the imbalance between the two arms of the trial is independent of age.

**Figure 2** Cumulative incidence of breast cancer in the study (●) and control (+) groups over 10 years of follow-up: (**a**) UICC clinical staging III, IV; (**b**) advanced disease as defined by Tabar *et al.*, 1985.

**Table V** Cases arising during the 3 year interval[a] between trial and service screens

| *Times from negative screen (months)* | *Number of cases* | *Rate/10,000 women-years* | *Proportion of control incidence* |
|---|---|---|---|
| 0-11 | 2 | 2.0 | 0.12 |
| 12-23 | 7 | 7.1 | 0.42 |
| 24-35 | 11 | 11.3 | 0.67 |
| 36 or over | 4 | 11.1 | 0.65 |
| Total | 24 | 7.3 | 0.43 |

[a]This is restricted to women who were still in regular screening in year 7 of the trial.

### Discussion

These results based on 10 years of follow-up confirm our earlier findings (Roberts *et al.*, 1990). There is a reduction in breast cancer mortality of around 18% in the total study population. This is not statistically significant and the confidence intervals are wide so that, by themselves, the results are inconclusive. They should be interpreted in context and are consistent with the consensus (Wald *et al.*, 1991) that mammographic screening reduces breast cancer mortality but by rather less than the 30% originally found in the HIP study (Shapiro *et al.*, 1982) and later by the Swedish two-counties trial (Tabar *et al.*, 1985). Other trials in Sweden have achieved smaller reductions, and the recent Swedish overview (Nystrom *et al.*, 1993) which includes all the Swedish trials estimates the reduction at 12 years of follow-up to be 22%; this is based on 882 deaths and is highly statistically significant. No other randomised trials of mammographic screening using an unscreened control group have been conducted outside Sweden since the HIP study, which was started in 1967. The Edinburgh trial, though numerically small, provides useful indications that the benefits observed in Sweden can be achieved elsewhere. This is important since the breast cancer experience in Sweden is very different; for example, cumulative mortality in the Edinburgh *study group* after 10 years (4.38/10,000 women-years) exceeded that in the Swedish two-counties *control group* after 12 years (4.15/10,000 women-years, from Nystrom *et al.*, 1993).

The TEDBC is larger than the Edinburgh trial but is a geographical comparison. Our data are not independent since the Edinburgh study group contributes to the TEDBC; the controls are, however, entirely distinct. Estimated reductions in breast cancer mortality for the TEDBC of 20% have recently been reported (UK Breast Cancer Detection Working Group, 1993); these are statistically significant but rely on difficult adjustments using pretrial breast cancer standardised mortality ratios (SMRs) for the geographical regions.

A number of design or execution problems for the study have been discussed previously (Roberts *et al.*, 1990) but should be noted here; these include the attendance rate, mammographic quality in the early years, loss of efficiency from cluster rather than individual randomisation and statistical power. All of these will have a conservative effect. Other potential sources of bias were, firstly, different use of adjuvant systemic therapy (Early Breast Cancer Trialists Collaborative Group, 1992) between the two groups and, secondly, differential errors of ascertainment of breast cancer deaths. We have demonstrated that the first does not apply and our methods of ascertainment avoid the latter.

Establishing whether death is attributable to breast cancer is difficult in a small number of cases. The review of a random sample of deaths with breast cancer underlined on the death certificate has confirmed previous findings: over-estimation of breast cancer as a cause of death of around 6% (Brinkley *et al.*, 1984; UK Breast Cancer Detection Working Group, 1991). The review of case notes reveals that small errors have occurred in the classification of the remaining deaths of breast cancer patients, but these data are compatible with other recommendations (Brinkley *et al.*, 1984) that analyses should use death certificate classifications of the underlying cause of death. Non-differential misclassification in the data will be a further source of conservative bias.

An unexpected consequence of the use of cluster sampling is the bias between the two arms of the trial, which is evidenced in the differences in all-cause mortality. This is in part explained by lower socioeconomic status in the control group (Alexander *et al.*, 1989). There is a body of evidence demonstrating that the direction of the association of socioeconomic status with breast cancer is opposite to that for all-cause mortality (Tomatis *et al.*, 1990; Scottish Breast Screening Programme, 1993). This suggests that the bias should be conservative, but our data have not permitted formal adjustment of the analyses for the effect of socioeconomic status. Despite the possibility of confounding in this study and inadequacy of SMR adjustment in the TEDBC, the point estimates from the two studies are very similar.

We conclude that our findings support the prevailing view that mammographic screening does reduce mortality from breast cancer – at least in women screened when 50 years and over (Wald *et al.*, 1991). Our data do not indicate that this age range fared better than the younger women.

There is currently no consensus on the merits of screening younger women (Beral, 1993; Elwood *et al.*, 1993; Fletcher *et al.*, 1993). The principal difficulty is that no trial of traditional design has been conducted with sufficient statistical power to analyse this age group separately. In addition, most authors fail to distinguish between the short and longer follow-up periods. For short follow-up periods (i.e. 7 years or less) all published trial results give point estimates of the relative risk which are either close to unity or exceed it (Shapiro *et al.*, 1982; Miller *et al.*, 1992; Nystrom *et al.*, 1993). This is supported by the Malmo trial (Andersson *et al.*, 1988) and the only relevant case–control study (Verbeek *et al.*, 1985). For longer follow-up periods the HIP trial reported reductions in breast cancer mortality for younger women (first screened when 40–49 years) which was eventually of the same magnitude as that in older women (Shapiro *et al.*, 1988) and, in one analysis (Chu *et al.*, 1988), achieved statistical significance. This is the only trial with follow-up exceeding 12 years but the Swedish trials have now reported a reduction of 13% after 12 years. Similar results have been reported by the Breast Cancer Demonstration Project, although this latter is neither population based nor randomised (Morrison *et al.*, 1988).

The Edinburgh results are consistent with the emerging consensus that modest reductions in breast cancer mortality are achieved for women first screened when under 50 years, but the benefits appear later than those for older women. At 7 years breast cancer mortality for women in the youngest age group was almost identical for the two arms of the trial. Now there is a reduction of 22% in the study group, but this does not approach statistical significance. These results with a randomised design and independent controls confirm the findings of the TEDBC [relative risk = 0.74 (0.54–1.0)] (UK Breast Cancer Detection Working Group, 1993). The data cannot be interpreted as evidence for population screening for younger women; they do, however, indicate that the optimal age to commence screening remains unknown; this is an important public health question which requires resolution by further randomised trials.

The classification of breast cancers by clinical stage and by pathological size and node status reveals an encouraging difference between study and control groups but is disappointing in one critical respect. This is the failure of screening to reduce the incidence of poor-prognosis disease as defined by Tabar *et al.* (1985) and, in particular, to achieve major reductions in the frequency of nodal metastases amongst the smaller cancers ($\leqslant$20 mm). Half of all invasive cancers in ever-screened women are poor prognosis compared with 33% in the Swedish two-counties trial (Tabar *et al.*, 1985). In both trials the percentages in the never-screened women (80% and 73% respectively) exceeded those in the controls, but the impact on the total study group is greater in Edinburgh on account of the lower attendance rate. The cumulative incidence of this poor-prognosis disease is not substantially reduced in the Edinburgh study group – although the results for the years 8–10 give preliminary indications that the curves may be diverging strongly. In the two-counties trial the changing incidence was reflected by the mortality curve (Tabar *et al.*, 1985), and subsequent analyses (Tabar *et al.*, 1992) have demonstrated that it is strongly predictive of mortality. Much of the failure of our mortality reduction to increase between 7 and 10 years of follow-up is likely to be explained by delay in this aspect of performance. Although we have detected large numbers of *in situ* cancer, this is predicted to have less impact on mortality than reducing the rate of small node-positive cancers (Tabar *et al.*, 1992). One of the strengths of the present trial database is that high achievement in pathological classifications of nodal status within one city has permitted an analysis which identifies the problem more clearly than is possible using tumour size alone (UK Breast Cancer Detection Working Group, 1993).

When service screening was introduced into Edinburgh we took the opportunity of designing a protocol which would enable us to report on the interval cancer rates over 3 years in regularly screened women; these should be predictive of eventual rates in the service programme with its 3 yearly schedule. The optimum interval between examinations is unknown; a randomised trial comparing 1 yearly intervals with 3 yearly ones is currently in progress (N. Day, personal communication) but will not report for several years. Meanwhile, the best method of evaluating the inter-screening interval is to compare the proportional incidence of interval cases across the period (Tabar *et al.*, 1987). The proportions we report are based on small numbers but exceed those for the two-counties trial in both the second (42% compared with 29%) and third (67% compared with 45%) year (Tabar *et al.*, 1987). This indicates, firstly, that the interval could be too long and should certainly not exceed 3 years – for the population or for individual women. Secondly, it may be further evidence that screening as practised during the trial lacked sensitivity for detecting biologically important cancers.

In conclusion, this trial has provided modest but important contributions to the overall scientific evaluation of mammographic screening. These include further evidence that screening – at least for women over 50 years – can reduce mortality from breast cancer by around 20%. With current standards of mammography and higher attendance rates larger reductions may be achievable. The extension of results from scientific trials to routine health care can be problematic, but current reports from UK service screening are encouraging (Chamberlain *et al.*, 1993; Scottish Breast Screening Programme, 1993). There is a need to determine the best age for screening to commence. Since the benefits of screening take at least 4 years to emerge, the present service screening programme though targeted at women of 50 years and over will have little impact on mortality from the disease in women aged 50–54 years despite the fact that 20% of all deaths of British women in this age group are due to breast cancer (OPCS, 1989–93).

Finally, two warning messages emerge from this study: firstly, follow-up and recall facilities must ensure that minimal numbers of women wait for more than 3 years between their invitations to screening. Secondly, screening targets must focus on detection of cancers before nodal metastases develop rather than relying on favourable size. It follows that it is essential to the monitoring of the UK service screening programme that histological evidence of node status is available on all cases arising in the target population.

The Edinburgh randomised trial was funded by the Cancer Research Campaign and the Chief Scientists Office (CSO) of the Scottish Home and Health Department. We thank the project committee for their advice and encouragement over the fieldwork period. The members of the committee were: Dr F.E. Alexander, Dr T.J. Anderson, Dr M.M. Andrew, Professor J.J.K. Best, Dr C. Brough, Dr W. Forbes, Professor A.P.M. Forrest (Chairman), Dr R. Gruer, Dr A. Huggins, Dr L. Kinlen, Dr A.E. Kirkpatrick, Dr N.B. Loudon, W. Lutz and Dr U. MacLean, Dr M.M. Roberts with Mr John Duncan and Dr Ian Sutherland. We especially wish to record the contribution made to this trial by Dr Maureen Roberts, who was director of the Edinburgh breast screening clinic from 1979 to her death in 1989. We also thank Dr Michael Ashley Millar and Dr Boyd Moir for help in coordinating the funding and Dr Helen Stewart for her assistance in the collection of recent data and classification of review causes of death. We acknowledge the assistance of the staff at the General Register Office, Scotland, the Lothian Health Board Primary Health Care Division and general practitioners in Edinburgh as well as our colleagues in the Screening Clinic and the University Departments of Public Health Science, Pathology and Surgery. Additional secretarial support was provided by the Leverhulme trust.

### References

ALEXANDER, F.E., ROBERTS, M.M., LUTZ, W. & HEPBURN, W. (1989). Randomisation by cluster and the problem of social class bias. *J. Epidermiol. Community Hlth*, **43**, 29-36.

ANDERSSON, I., ASPERGREN, K., JANZON, L. & 6 others (1988). Mammoraphic screening and mortality from breast cancer: the Malmo trial. *Br. Med. J.*, **297**, 943-948.

BERAL, V. (1993). Breast cancer: mammographic screening. *Lancet*, **341**, 1509-1510.

BRINKLEY, D., HAYBRITTLE, J.L. & ALDERSON, M.R. (1984). Death certification in cancer of the breast. *Br. Med. J.*, **289**, 465-467.

CHAMBERLAIN, J., MOSS, S.M., KIRKPATRICK, A.C., MITCHELL, M. & JOHNS, L. (1993). National Health Service breast screening programme results for 1991-2. *Br. Med. J.*, **307**, 353-356.

CHU, K.C., SMART, C.R. & TARONE, R.E. (1988). Analysis of breast cancer mortality and stage distribution by age for the Health Insurance Plan clinical trial. *J. Natl Cancer Inst.*, **80**, 1125-1131.

EARLY BREAST CANCER TRIALISTS COLLABORATIVE GROUP (1992). Systematic treatment of early breast cancer by hormonal, cytotoxic or immunotherapy. *Lancet*, **i**, 1-17.

ELWOOD, J.M., COX, B. & RICHARDSON, A.K. (1993). The effectiveness of breast cancer screening in young women. *Curr. Clin. Trials*, **2**, 227-287.

FLETCHER, S.W., BLACK, W., HARRIS, R., RIMER, B.K. & SHAPIRO, S. (1993). Report of the international workshop on screening for breast cancer. *J. Natl Cancer Inst.* (in press).

FORREST, A.P.M. (CHAIRMAN) (1987). *Breast Cancer Screening*, report to the Health Ministers of England, Wales, Scotland and Northern Ireland by a working group. HMSO: London.

MILLER, A.B., BAINES, C.J., TO, T. & WALL, C. (1992). Canadian National Breast Screening Study: I. Breast cancer detection and death rates among women 40 to 49 years. *Can. Med. Assoc. J.*, **147**, 1459-1488.

MORRISON, A.S., BRISSON, J. & KHALID, N. (1988). Breast cancer incidence in the breast cancer demonstration project. *J. Natl Cancer Inst.*, **80**, 1540-1547.

NYSTROM, L., RUTQVIST, L.E., WALL, S. & others (1993). Breast cancer screening with mammography: an overview of the Swedish randomised trials. *Lancet*, **341**, 973-978.

OPCS MORTALITY STATISTICS: CAUSE. (1985). Series DH2 No. 11. HMSO: London.

OPCS CANCER STATISTICS: REGISTRATIONS. (1989-93). Series HBI Nos. 16-20 HMSO: London.

ROBERTS, M.M., ALEXANDER, F.E., ANDERSON, T.J. & 7 others (1984). The Edinburgh randomised trial of screening for breast cancer: description of method. *Br. J. Cancer*, **47**, 1-6.

ROBERTS, M.M., ALEXANDER, F.E., ANDERSON, T.J. & 9 others (1990). Edinburgh trial of screening for breast cancer: mortality at seven years. *Lancet*, **335**, 241-246.

SCOTTISH BREAST SCREENING PROGRAMME (1993). *Scottish Breast Screening Programme Report, 1993*. ISD Publications: Edinburgh.

SHAPIRO, S., VENET, W., STRAX, P. & ROESER, R. (1982). Ten-to-fourteen year effect of screening on breast cancer mortality. *J. Natl Cancer Inst.*, **69**, 349-355.

SHAPIRO, S. (1988). *Periodic Screening for Breast Cancer: The Health Insurance Plan Project and its Sequelae, 1963-1986*. Johns Hopkins University Press: Baltimore.

TABAR, L., FAGERBERG, C.J.G., GAD, A. & 8 others (1985). Reduction in mortality from breast cancer screening with mammography. *Lancet*, **i**, 829-832.

TABAR, L., FAGERBERG, C.J.G., GAD, A. & 2 others (1987). What is the optimal interval between mammographic screening examinations? An analysis based on the latest results of the Swedish two-county breast cancer screening trial. *Br. J. Cancer*, **55**, 547-551.

TABAR, L., FAGERBERG, G., DUFFY, S.W., DAY, N.E., GAD, A. & GRONTOFT, O. (1992). Update of the Swedish two-county program of mammographic screening for breast cancer. *Radiol. Clin. N. Am.*, **30**, 187-210.

TOMATIS, L., AITIO, A., DAY, N.E. & 5 others (eds) (1990). *Cancer: Causes, Occurrence and Control*. IARC: Lyons.

UK BREAST CANCER DETECTION WORKING GROUP (1981). Trial of early detection of breast cancer: description of method. *Br. J. Cancer*, **44**, 618-623.

UK BREAST CANCER DETECTION WORKING GROUP (1993). Breast cancer mortality after 10 years in the UK trial of early detection of breast cancer. *Breast*, **2**, 13-20.

UK BREAST CANCER DETECTION WORKING GROUP (1991). Verification of the cause of death in the trial of early detection of breast cancer. *Br. J. Cancer*, **64**, 1151-1156.

VERBEEK, A.L.M., HENDRIKS, J.H.C.L., HOLLAND, R., MRAVUNAC, M. & STURMANS, F. (1985). Mammographic screening and breast cancer mortality: age-specific effects in the Nijmegen project, 1975-82. *Lancet*, **i**, 865-856.

WALD, N., FROST, C. & CUCKLE, H. (1991). Breast cancer screening: the current position. *Br. Med. J.*, **302**, 845.

WILLIAMS, D.A. (1982). Extra-binomial variation in logistic linear models. *Appl. Stat.* **31**, 144-148.